# NIST Crystallographic Databases for Research and Analysis

**DOI:** 10.6028/jres.101.028

**Published:** 1996

**Authors:** Alan D. Mighell, Vicky Lynn Karen

**Affiliations:** National Institute of Standards and Technology, Gaithersburg, MD 20899-0001

**Keywords:** crystalline materials, crystallography, database, electron diffraction, lattice, lattice relationships, materials design, NIST Crystal Data, phase identification

## Abstract

The NIST Crystal and Electron Diffraction Data Center builds a comprehensive database with evaluated chemical, physical, and crystallographic information on all types of well-characterized substances. The data are evaluated and standardized by specially designed computer programs as well as by experts in the field. From its master database, the Data Center produces NIST Crystal Data and an Electron Diffraction Database with over 220 000 and 81 000 entries, respectively. These distribution databases are made available to the scientific community via CD-ROM, scientific instruments and online systems. In addition, the Data Center has developed theory and software that can be used for establishing all types of lattice relationships, for the determination of symmetry, for the identification of unknowns using lattice matching techniques, and for data evaluation.

## 1. Introduction

The NIST Crystal and Electron Diffraction Data Center is concerned with the collection, evaluation and dissemination of data on solid-state materials. The Data Center maintains a comprehensive master database with information on all types of well-characterized crystalline compounds. The entries cover all categories of materials including inorganics, organics, organometallics, metals, intermetallics and minerals. Each entry contains chemical, physical and crystallographic information such as the author’s cell, Crystal Data cell, reduced cell, crystal system, space group, Pearson’s symbol, chemical name and formula, empirical formula, chemical class, density, literature references, cross references to other databases, etc. A principal focus of the Data Center has been to standardize and evaluate the data by specially designed computer programs as well as by experts in the field.

Currently, the master database is growing at a rate of approximately 15 000 new entries each year. From this master database, the Data Center produces NIST Crystal Data [1] with over 220 000 entries and the Electron Diffraction Database [2] with over 81 000 entries. These databases are periodically updated and made available to the scientific community via CD-ROM, scientific instruments and online systems. Along with the databases, the Data Center has developed theory and software that can be used for establishing all types of inter-and intralattice relationships, for the determination of symmetry, for the calculation of reduced and standard cells, for the determination of derivative cells, and for identification of unknowns via lattice matching techniques. These distribution databases along with the associated scientific software represent valuable tools for research and analysis.

## 2. NIST Crystal Data

The information contained in NIST Crystal Data is of interest to scientists in many disciplines, as it includes data from the entire spectrum of well-characterized materials. These disciplines include analytical chemistry, materials science, crystallography, mineralogy, ceramics, metallurgy, organic chemistry, biochemistry, physical chemistry, inorganic chemistry, and physics, among others. Due to its comprehensive nature, NIST Crystal Data can be cross-referenced to other databases whereby additional data on a specific material can be retrieved. There are two fundamental ways that large crystallographic databases can be used. As a source of critically evaluated data, NIST Crystal Data can be used as a *basis* for scientific research, or as an *aid* to scientific research. For example, space group statistics provide the researcher with a summary of what happens in nature and, as such, may ultimately provide the conceptual link required for the *a priori* prediction of the crystal structure from the molecular shape. Likewise, statistics derived from the database can assist scientists in the preparation of new materials, as in the example of optical materials where knowledge of space group frequencies can prove invaluable. Some typical searches are summarized in Table 1.

### 2.1 Searching the Database

Since the data in NIST Crystal Data are evaluated, standardized, and organized, it is possible to carry out searches on all the data parameters using independently written computer programs. However, it is most convenient to put NIST Crystal Data under a database management system that has been coupled with specially designed scientific software.

A general search strategy based on Boolean operations consists of three basic steps: 1) the search question is analyzed and framed into the form of several discrete search parameters; 2) for each parameter, the database is searched and the subset of data consisting of potential answers is saved; and, finally, 3) the subsets of data are intersected using Boolean ‘AND,’ ‘OR,’ and ‘NOT’ operations. If the resulting data subset is not sufficiently specific, additional search parameters may be formulated to obtain the desired results. To obtain all solutions of potential interest, the search parameters should be as nonrestrictive as possible. For example, a simple search of element types present in the formula, rather than on the exact formula, is the search most commonly used in solving problems directed to the NIST Crystal and Electron Diffraction Data Center. Even if all the search parameters are limited or restricted in some way (e.g., a partial chemical analysis, a single cell parameter, a few *d*-spacings, or an approximate density), it is usually possible to solve a problem.

### 2.2 Lattice Matching Algorithms

There are two basic strategies that can be used to determine whether two unit cells define the same or related lattices. In the first method, lattice relationships are deduced by comparing standard (i.e., most commonly, reduced) cells and standard derivative cells. In the second method, one determines the group of matrices relating two lattices using converse-transformation analysis [3–5]. The nature of the relationship is deduced by analyzing the matrices.

The present version of the search program designed at NIST uses the first strategy. The experimentally determined unit cells for both the known and unknown compounds are converted to standard reduced cells. The reduced cell parameters are then compared; if the reduced cells are the same, then the original cells define the same lattice. Reduction is a mathematical procedure that leads to a unique cell in all cases, provided there is no experimental error in the unit cell parameters. In practice, two experimentally determined cells defining the same lattice will always give the same *a*, *b*, *c* within experimental error for the reduced cell, but the reduced cell angles may differ. Consequently, in order to obtain all potential matches, it is prudent to match only cell edges and cell volume of the unknown reduced cell against the known cells in NIST Crystal Data. It is possible that every match obtained in this way does not define the same lattice. For those cases in which more than one match of *a*, *b*, *c*, *V* occurs, knowledge of the empirical formula or element-types present is almost always sufficient to eliminate unwanted matches.

In sharp contrast to presently employed procedures that are based on standard reduced cells, lattice matching can be carried out using a powerful new mathematical procedure based on the converse-transformation operator [3–5]. In this approach, standard cells are not required. Using an algorithm based on this operator, matrices relating any two lattices can be readily determined. The precise nature of the lattice relationship is then deduced from the matrices. Consequently, the experimentalist can determine if two lattices are the same or if they are in a derivative lattice relationship. This approach is far superior to all approaches based on matching standard cells. With converse-transformation, the mathematics and algorithms used to analyze lattice relationships provide both the conceptual and practical framework required to rigorously analyze experimental data regardless of the magnitudes of the experimental error. Converse transformation forms the basis for the algorithm presently used in the lattice relating routines in NIST*LATTICE [5]. With this software, the experimentalist can establish the relationship between any two lattices. In the future, it will be the basis of our identification code used to match unknowns against the database.

### 2.3 Use of the Database for Materials Characterization and Identification

NIST Crystal Data represents a powerful resource for the identification of unknown materials. Research in our laboratory has shown that the lattice (as defined by a unit cell) is highly characteristic of a compound and, like a fingerprint, may be used for identification. When coupled with limited chemical information, this method of identification yields unique results.

#### 2.3.1 Identification Strategy

**Fig. 1:**
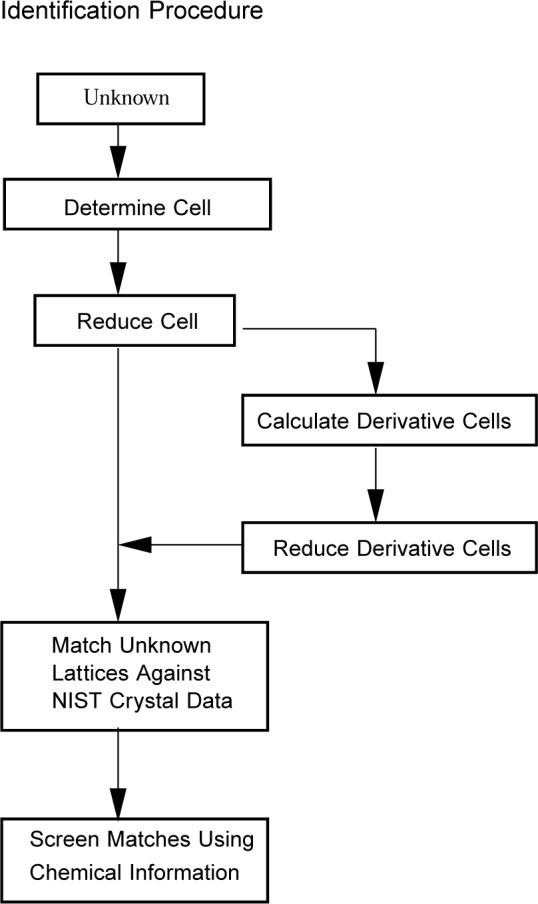
In this scheme, a unit cell that defines the lattice is determined on a diffractometer and the identification is carried out by lattice matching against NIST Crystal Data. The matches are screened using chemical information such as element-type data.

The basic identification strategy is to check the lattice of the unknown against all lattices in the database for a match and then to exclude unwanted matches on the basis of chemical information. A summary of the lattice matching procedure is presented in Fig. 1. In this scheme, an unknown crystal is selected and mounted on a single-crystal diffractometer and the unit cell is determined and reduced. The reduced cell is then checked against the file of known materials. If desired, one calculates derivative lattices which are also reduced and checked against the file of knowns. In the above scheme, the cell defining the lattice was determined on a single crystal diffractometer. It can also be obtained from films, from lattice data collected on an electron microscope, and by indexing *d*-spacings determined on a powder diffractometer. An example of the integration of the database and search software within the Siemens’ x-ray diffractometer^1^ is described in this Special Issue in the manuscript entitled “Using the NIST Crystal Data within Siemens’ Software for Single Crystal and SMART CCD Diffractometers” [6].

Experience with practical problems has shown that identification by matching reduced cells is very straightforward and reliable when the correct cell of the lattice has been determined. However, a material can often be identified even if an error has been made in which a derivative cell of the unknown has been found. For example, a derivative cell of the correct lattice would be determined if the cell centering is missed when indexing a powder pattern or if rows of spots are missed in reciprocal space when using single-crystal methods. One can identify an unknown, in spite of errors of this type, by systematically calculating derivative supercells and sub-cells and looking for matches in NIST Crystal Data. Checks for the 55 supercells of 2, 3, 4 times the volume of the unknown reduced cell and the 55 subcells of 1/2, 1/3, 1/4 times the volume are routinely made. The ability to check routinely for matches of 110 extra derivative cells for each unknown is possible because of the efficiency of the computer program and the selectivity of the method. Further details on lattice matching and on the calculation of derivative lattices have been published in Acta Crystallographica [7] and in an NBS Technical Note [8].

#### 2.3.2 Lattice Matching using Electron Diffraction Data

Both NIST Crystal Data and the Electron Diffraction Database contain unit cell and element-type data. There are a variety of approaches that may lead to the determination of a unit cell on the Analytical Electron Microscope suitable for routine identification. For example, Goehner and Michael [9] have developed a technique whereby they can obtain high quality backscattered electron Kikuchi patterns. If a unit cell can be extracted from such data, then it will be possible to routinely identify unknowns from this type of data, thereby creating a powerful analytical procedure for phase characterization. Using lattice matching algorithms based on the converse transformation operator, it is possible to match reliably an unknown cell, even with large errors, against the database. Recently, a new phase identification procedure for electron diffractionists that utilizes this approach has been described [4, 10].

#### 2.3.3 Prevention of Duplicate Publication

As an integral part of the editorial review procedure, the International Union of Crystallography (IUCr) journals are using screening procedures to prevent the duplicate publication of data on the same crystalline phase. Recently, NIST Crystal Data and NIST search software were installed in the Technical Office of Acta Crystallographica. Using lattice-matching techniques, each incoming structure can routinely be checked against the database to see if the compound or a related structure has been previously studied.

### 2.4 Analytical Examples

The single-crystal diffractometer, which started out primarily as a research instrument, is finally making its transition to widespread use in the analytical laboratory. Three relatively recent developments have given the lattice method for compound identification great potential as a routine analytical tool. First, the integration of NIST Crystal Data with commercially available diffractometers makes the method readily available, automated, and easy to use. Second, the lattice method of identification is superior to existing methods (e.g., the powder method) because of its high selectivity and reliability even with very large errors on the lattice parameters. Finally, new mathematical techniques permit fast and effective identification of the direct lattice in addition to enabling one to establish many other types of important interlattice relationships via derivative lattice and converse-transformation theory.

**Fig. 2:**
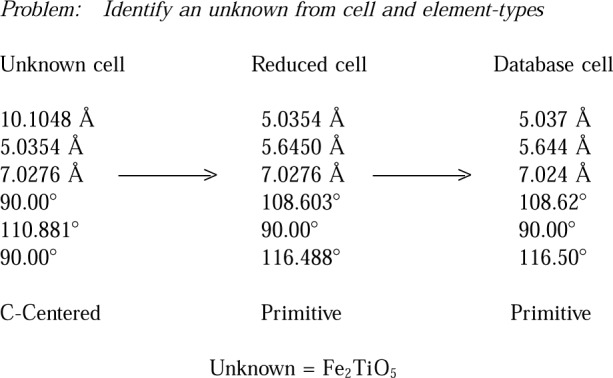
The reduced cell of the unknown matched the database for Fe_2_TiO_5_. As the unknown was known to contain Fe, Ti, and O, it was deduced to be identical to the material in the database.

**Fig. 3:**
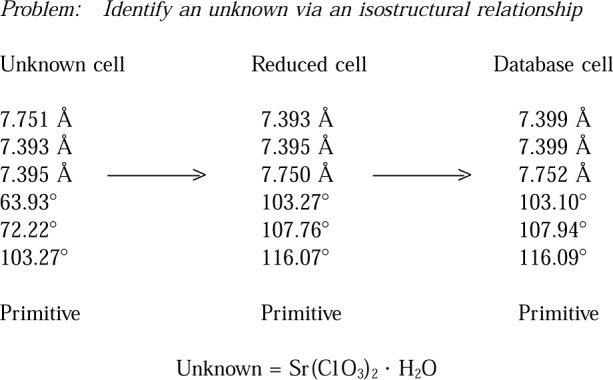
The reduced cell of the unknown matches the database cell for Ba(ClO_3_)_2_ · H_2_O. Since the unknown contains Sr, it is isostructural with the compound in the database.

**Fig. 4:**
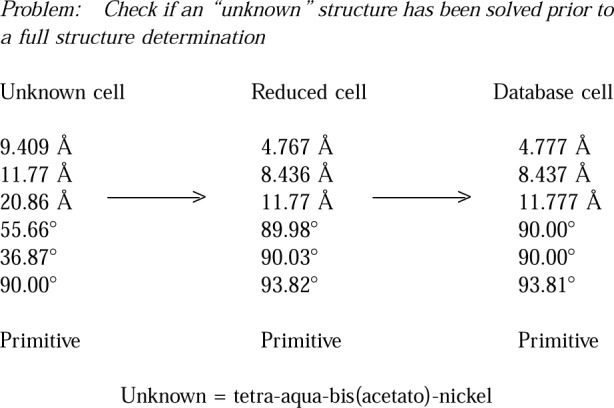
The unknown reduced cell matched the database cell for the compound tetra-aqua-bis(acetato)-nickel. Consequently, a redetermination of the structure was not carried out.

**Fig. 5:**
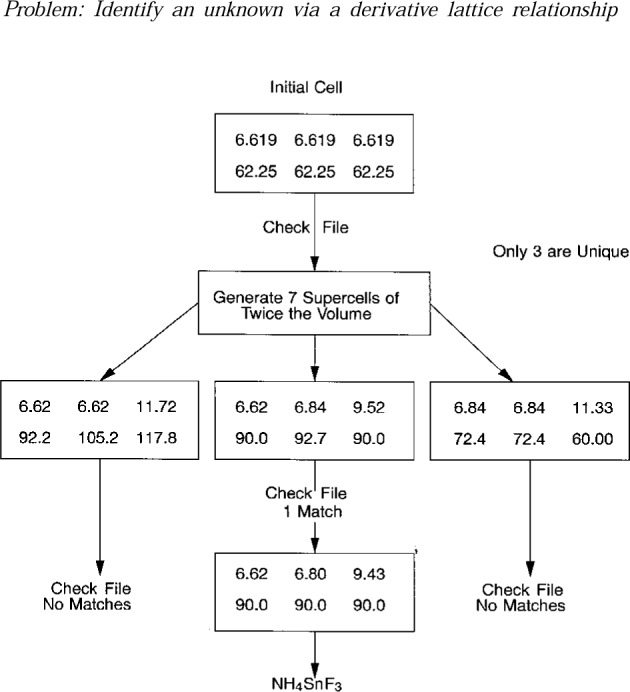
A derivative supercell of the unknown matched the database cell for NH_4_SnF_3_. In this case, the initial unknown cell is correct whereas the database cell is a supercell. The cell edges are in Ås and the angles in degrees.

**Fig. 6:**
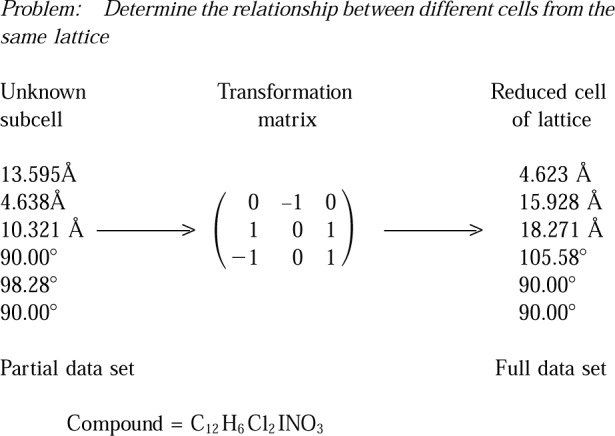
The initial cell determined on the diffractometer was a sub-cell of the correct lattice. Structure solutions on the basis of the first and second cells produced disordered and ordered structures, respectively.

Lattice matching against the database can be used to solve many problems in the academic and industrial environment. For example, in his paper on “Conventional and Eccentric Uses of Crystallographic Databases in Practical Materials Identification Problems,” J. Kaduk [11] discusses how NIST Crystal Data can be used in materials identification in the industrial environment. Additional practical examples of identification by lattice matching techniques are illustrated in Figs. 2–6.

An example of an identification based on lattice parameters and some chemical information is illustrated in Fig. 2. A crystal which was known to contain Fe, O, and Ti was mounted on a single-crystal x-ray diffractometer and a C-centered monoclinic cell was determined. The cell was transformed to a primitive cell and reduced. The reduced cell was then checked against NIST Crystal Data. The sample was found to be Fe_2_TiO_5_ by a direct match of the reduced cell parameters. As noted above, a match of the lattice and element types uniquely identifies or characterizes a material.

Figure 3 demonstrates an identification that was made on the basis of the cell parameters and limited chemical data. It was known that the compound contained strontium. First, the initial cell was determined on an automated single-crystal diffractometer. Next, the reduced cell was calculated and checked against NIST Crystal Data. A direct match was obtained. The unknown was deduced to be Sr(ClO_3_)_2_ · H_2_O because it was isostructural with the barium analog in the file of knowns.

Before collecting a full set of diffraction data on an “unknown” crystal, one should routinely check the database to see whether the structure of the compound or a closely related material has already been determined. As Fig. 4 shows, this check is carried out as soon as any primitive cell of the lattice has been determined. The experimental cell was transformed to the reduced cell which matched exactly an entry in NIST Crystal Data. The compound was identified and the full structure determination was halted. This identification procedure is now a standard option in the automated procedure associated with the Siemens’ diffractometer [6].

Figure 5 shows how an identification can be made by using a derivative lattice procedure. When using powder methods, a supercell in direct space may be determined if one does not find the smallest cell consistent with a set of *d*-spacings. In single-crystal work, a subcell in direct space is determined if reciprocal lattice nodes are missed on a diffraction photograph or a diffractometer. In either case, it is possible to systematically calculate derivative cells and identify the material. Here, the unknown cell was correct and the known cell was a super-cell. The initial cell and space group were determined on a single-crystal diffractometer and a full set of diffraction data was collected. The empirical formula was known. When checked against the database of known compounds, there were no matches. Next, the seven supercells of twice the volume were calculated; only three were unique because the initial cell is metrically rhombohedral. One of the three calculated supercells matched a cell in NIST Crystal Data. The known cell, which was a supercell of the correct cell, had been determined from powder data only. Nevertheless, it was possible to verify the correct composition and to find appropriate literature references.

Figure 6 illustrates a case in which a structure was initially “solved” using a derivative cell. Using a subcell, the data were collected and the structure was determined. After analyzing the resulting disordered structure, the structure solution was repeated. The unit cell was determined a second time and was found to have twice the volume of the initial cell. When the data were re-collected and the structure was solved on the basis of the second cell, the disorder disappeared.

It is not uncommon for a derivative subcell to be determined in routine structure work. Incorrect structure solutions based on a derivative subcell often reveal “disorder.” Another example of this is given in this issue by Richard Harlow in his paper “Troublesome Crystal Structures: Prevention, Detection and Resolution [12].” Fortunately, in cases in which a sublattice has unknowingly been determined, the experimentalist can still make a positive identification by routinely calculating and matching derivative lattices.

## 3. Applications of the Electron Diffraction Database

The Electron Diffraction Database enables the experimentalist to identify materials from extremely small crystalline samples. This capability is important as many disciplines in the materials sciences are carrying out research on the microscopic level. This Database contains all the data required to identify unknowns using experimental data collected on the Analytical Electron Microscope (AEM). It is comprehensive in nature and contains data on all classes of inorganic materials. For each material, calculated *d*-spacings are included if the cell is known, otherwise observed *d*-spacings are used. With the AEM, the experimentalist can readily determine the elements present in an unknown as well as a number of interplanar spacings. By matching this observed data against the Electron Diffraction Database, one can identify the sample or obtain a limited set of possibilities.

Even though it was originally designed for the electron diffraction community, this database has important applications in other fields in which interplanar spacings are measured, calculated or analyzed. As the database includes calculated *d*-spacings for all entries, a great variety of inter- and intralattice relationships can be established. For example, the interplanar spacings obtained from widely used powder diffraction experiments can be used to identify unknown samples. When the Electron Diffraction Database and NIST Crystal Data are used in conjunction with each other, many complex research and analytical problems can be solved. For example, in the solution of structures via powder profile analysis, NIST Crystal Data can be used to identify the main phase while the Electron Diffraction Database can be used to identify potential impurities from the “extra” lines.

## 4. Dissemination

NIST Crystal Data and the Electron Diffraction Database are made available to the scientific community through computer oriented modes of dissemination including CD-ROM/PC, scientific instruments, and online systems. Each of these distribution modes is briefly discussed below:

### 4.1 CD-ROM/PC

Both databases are distributed on CD-ROM or disc along with search software. Searches can be carried out at one’s desk using a personal computer. All key parameters in NIST Crystal Data are searchable using Boolean logic (AND, OR, NOT). Search parameters include the literature cell, Crystal Data cell, reduced cell, space group, crystal system, chemical formula, empirical formula, references, density, etc. Unknowns can be identified against NIST Crystal Data using lattice/formula matching techniques developed at NIST. For the Electron Diffraction Database, specially designed search software has been developed that permits identification based on *d*-spacing/element-type matching strategies. Comprehensive search software is also available through private vendors.

### 4.2 Analytical Electron Microscope

The Electron Diffraction Database has been built into the work stations associated with commercial Analytical Electron Microscopes. Unknown phases can be identified as soon as elemental and *d*-spacing data have been recorded.

### 4.3 Analytical X-Ray Instruments

NIST Crystal Data and search theory have now been integrated into commercial single-crystal x-ray diffractometers. As an integral part of every structure determination, the database can be routinely searched. This step is carried out in the initial stage of a structure determination as soon as three vectors defining the lattice have been determined. Consequently, the investigator will immediately be aware if his sample is “new” or if the compound is related in some way to an existing material. Unnecessary structure determinations will be prevented. More importantly, the database-instrument combination creates a new analytical tool for materials characterization. With this new automated tool, the identification of an unknown crystal can be carried out within a few minutes.

### 4.4 Online Searching

NIST Crystal Data and the Electron Diffraction Database can be accessed using online search systems. Such systems consist of the database along with search and analysis software. Search tools that are based on Boolean logic are commonly employed. An example of such a system is CRYSTDAT which is made available to the worldwide scientific community by the Canada Institute of Science and Technology. Online search systems are ideal for analytical purposes, materials research and statistical studies. Because of the rapid evolution of international communication networks, online systems are destined to play an increasingly important role in the delivery of scientific information.

## 5. Future Directions

The Data Center is concerned with database building, the critical evaluation of data, and dissemination. All three functions will undergo rapid changes in the future. With respect to database building, it is projected that the master database will grow at the rate of at least 15 000 entries per year. Over the years, the Data Center has developed close collaborative ties with other crystallographic data centers and with the International Union of Crystallography. Through data exchange agreements, such collaboration has helped to assure that NIST Crystal Data is complete with respect to all categories of data. We look forward to a future with continued and enhanced cooperation in the crystallographic community.

For the critical evaluation of data, future trends will include the development of theory and the design of algorithms and strategies to analyze sets of data within and between databases. Algorithms will be designed to look for patterns of errors that result from faulty experimental procedures. Once such patterns are found, experimental procedures can be fixed so that errors are prevented at the earliest possible stage in the data process.

Future directions in dissemination will include the development and implementation of state-of-the-art PC search systems as well as increasingly sophisticated on-line systems. In addition, users are requesting that databases with information on crystalline materials be searched in conjunction with each other using standardized and simple to use search tools.

## 6. Availability: Databases and Software

For information concerning the Databases, contact Alan Mighell or Vicky Lynn Karen of the NIST Crystal and Electron Diffraction Data Center, Materials Science and Engineering Laboratory, National Institute of Standards and Technology, Gaithersburg, MD 20899 (phone: 301-975-6255, fax: 301-926-0416).

To obtain information on converse-transformation analysis and scientific software for analyzing lattice relationships (NIST*LATTICE), contact Vicky Lynn Karen (email: karen@tiber.nist.gov, phone: 301-975-6255).

## Figures and Tables

**Table 1:** Typical searches using NIST Crystal Data

1)	Find all compounds containing N, Cu, H, Br, and O.
2)	Find all nickel containing compounds with lattice parameters: 5.31 Å, 4.85Å, 6.26 Å, 68.0°, 69.8°, 91.0°.
3)	Find all references to steroids in the monoclinic space group P2_1_ that have four molecules in the asymmetric unit (to search for hydrogen bonds between molecules not symmetrically related).
4)	Find the cell parameters and space groups for all compounds containing Fe and O and only three elements.
5)	Find polymorphs of the amino acid glycine. Find all references to alanine for which the full structure has been determined.
6)	Find all materials with a density between 3.0 g/cm^3^ and 4.0 g/cm^3^ with a cell volume in the range 900 Å to 1000 Å, and with only Ni, Se, and one other chemical element in the formula.
7)	Find all organic substances that have the character string “ene” in the chemical name, that have three Cu atoms in the molecule, and that crystallize in the triclinic or monoclinic crystal systems.
8)	Find all compounds containing Pm and O with a cell parameter (from electron diffraction) in the range 3.5 Å to 3.7 Å.
9)	Find compounds with specified lattice parameters, space group, and chemistry to aid in the design of lasers and semiconductors.
10)	Find all lattices and derivative lattices related to the lattice of a known superconductor.
11)	Find space group frequencies for all compounds. Find frequencies for special categories of materials such as binary rare-earth oxides.
12)	Find the least-populated space groups for organic compounds.
13)	Find the number of compounds that crystallize in space group C2/c that have rhombohedral metric symmetry.
14)	Find all molecular compounds crystallizing in space group P2_1_/c with *Z* greater than 4.

## Footnotes

1 Certain commercial equipment, instruments, or materials are identified in this paper to foster understanding. Such identification does not imply recommendation or endorsement by the National Institute of Standards and Technology, nor does it imply that the materials or equipment identified are necessarily the best available for the purpose.
